# The Role of Gamma Delta T Lymphocytes in Physiological and Pathological Condition—Focus on Psoriasis, Atopic Dermatitis, Autoimmune Disorders, Cancer and Lymphomas

**DOI:** 10.3390/ijms25147960

**Published:** 2024-07-21

**Authors:** Joanna Chojnacka-Purpurowicz, Agnieszka Owczarczyk-Saczonek, Bogusław Nedoszytko

**Affiliations:** 1 Department of Dermatology, Sexually Transmitted Diseases and Clinical Immunology, The University of Warmia and Mazury, 10-719 Olsztyn, Poland; agnieszka.owczarczyk@uwm.edu.pl; 2 Department of Medical Laboratory Diagnostics–Fahrenheit Biobank BBMRI.pl, Medical University of Gdansk, 3A M. Skłodowskiej-Curie Street, 80-210 Gdansk, Poland; bned@gumed.edu.pl; 3 Molecular Laboratory, Invicta Fertility and Reproductive Center, 81-740 Sopot, Poland

**Keywords:** psoriasis, pathogenesis of psoriasis, T lymphocytes, T-memory cells, gamma delta T lymphocytes, cytokines, atopic dermatitis, lymphoma, cancer

## Abstract

**Gamma delta** (γδ) T cells are a heterogeneous population of cells that play roles in inflammation, host tissue repair, clearance of viral and bacterial pathogens, regulation of immune processes, and tumor surveillance. Recent research suggests that these are the main skin cells that produce interleukin-17 (I-17). Furthermore, γδ T cells exhibit memory-cell-like characteristics that mediate repeated episodes of psoriatic inflammation. γδ T cells are found in epithelial tissues, where many cancers develop. There, they participate in antitumor immunity as cytotoxic cells or as immune coordinators. γδ T cells also participate in host defense, immune surveillance, and immune homeostasis. The aim of this review is to present the importance of γδ T cells in physiological and pathological diseases, such as psoriasis, atopic dermatitis, autoimmune diseases, cancer, and lymphoma.

## 1. Introduction

Gamma delta (γδ) T cells are a type of T cell that constitutes approximately 1–5% of circulating lymphocytes in the blood [1]. γδ T cells are present both in the epidermis and dermis [2]. Recent studies have shown that γδ T cells may constitute up to 50% of T cells in epithelial tissues and mucous membranes [3,4].

Most T cells have alpha beta receptor (TCR) T cells (Tαβ) composed of two chains of glycoproteins, called the α (alpha) and β (beta) chains of the TCR. In contrast, γδ T cells have a TCR that consists of one γ (gamma) and one δ (delta) chain [5].

γδ T cells are derived from the same progenitor as αβ T cells; however, unlike αβ T cells, which leave the thymus as naive T cells, γδ T cells acquire effector function already in the thymus, linking the innate and adaptive immune systems by participating in various physiological and pathological processes [6].

γδ T cells develop in the thymus during the fetal period [7]. During ontogeny in the thymus, γδ T cells develop before αβ T cells. In healthy adults, they constitute a small amount of all circulating lymphocytes with a predominantly CD4/CD8 double-negative phenotype [6]. γδ T cells can be classified based on TCR Vδ expression. There are subtypes Vδ1, Vδ2, Vδ3, and Vδ5 [7]. Vδ1 T cells are found in the intestinal mucosa, skin, liver, lungs, and spleen. Vδ2 T cells are found mainly in peripheral blood, while Vδ3 T cells constitute 0.2% of circulating cells and are found mainly in the liver and intestines.

The γδ T cell lineage is influenced by numerous signals, including TCR signal strength, notch and IL-7R signaling, and the presence of double-positive thymocytes. Strong TCR signals promote the development of γδ T cells, whereas relatively weak TCR signals generate αβ T lineage cells [8].

γδ T cells are innate lymphocytes, unrestricted by the major histocompatibility complex (MHC). They have a larger number of antigen receptors compared with cells with αβ T cells [5].

## 2. Activation of γδ T Cells

αβ T cell activation and cytokine production rely on three successive signals: TCR ligation, stimulation of costimulatory molecules, and cytokine signaling. Costimulation is generated by the interaction between molecules such as CD28 on αβ T cells and ligands, such as CD80 and CD86. The activation of γδ T cells is less clear. Although γδ T cells express the TCR, it does not engage MHC–antigen complexes in the same way as T cells [3]. However, there are some similarities to the activation of αβ T cells. The entire repertoire of antigens recognized by γδ T cells is still unknown; however, it is known that the γδ TCR is required for antigen recognition [9].

Unlike αβ T cells, the lack of MHC class II or β2 microglobulin does not affect the development of γδ T cells. γδ T cells emerge from the thymus as mature γδ T cells and do not require TCR signaling for their differentiation and function. However, a recent report demonstrated that TCR-mediated activation is critical for IL-17A production in Dendritic Epithelial T Cells (DETCs) during the wound healing response [10].

Unlike αβ T cells, γδ T cell receptors recognize antigens directly, without the need for processing and presentation by antigen-presenting cells (APCs) [11].

The most key discrepancy between αβ and γδ T cells lies in their activation mechanisms and antigen presentation abilities. αβ T cell activation requires antigen recognition and costimulation to mount immune responses, whereas γδ T cells can be activated by a single signal, such as a phosphoantigen. A specific subset of human γδ T cells, Vδ2 T cells, assume the role of APCs. This unique feature makes γδ T cells key regulators of immune function [12]. γδ T cells have been shown to recognize MHC class I-related molecules, MICA and MICB, which can be induced by infection, injury, or cellular transformation. These molecules are stimulatory ligands for the NK cell receptor, NKG2D, which is also expressed in γδ T cells [13].

Vγ9Vδ2 T cells participate in defense against malignant cells by detecting phosphoantigen, isopentenyl pyrophosphate (IPP), or pathogen molecules. Ligand recognition by Vγ9Vδ2 T cells requires butyrophilin (BTN3A1) and BTN2A1. Vγ9Vδ2 T cells sense increased levels of intracellular phosphoantigen, causing them to activate and kill target cells [14].

The chemokine receptor CCR6 and the transcription factor RORγt are associated with the development and recruitment of CD4 + Th17 cells and are also expressed in splenic γδ T cells. γδ T cells located in the skin have been shown to constitutively express CCR6 and RORγt [15].

Costa et al. demonstrated that neutrophils inhibit γδ T cell functions by producing reactive oxygen species (ROS). Spleen tyrosine kinase (Syk), a nonreceptor tyrosine kinase, transmits signals from various immune receptors in neutrophils. Syk is a key signaling molecule involved in modulating the ability of neutrophils to inhibit γδ T cell proliferation through ROS production [16]

## 3. The Role of γδ T Lymphocytes in Physiological Processes

γδ T cells are heterogeneous populations of cells that play a role in inflammation, host tissue repair, clearance of viral and bacterial pathogens, regulation of immune processes, and tumor surveillance [5,17].

γδ T cells have the properties of innate immune cells [18]. They play an important role in the response to pathogens in the skin. γδ T cells produce IL-17 and IL-22 when stimulated with IL-23, triggering the production of antimicrobial peptides by keratinocytes, as well as the migration of neutrophils to the site of infection [1]. γδ T cells act as the primary defense against pathogen invasion, especially in early life. They secrete chemokines that attract neutrophils to the site of inflammation and help clear pathogens [10].

Depending on the location and inflammatory signals present in the tissue microenvironment, γδ T cells exhibit phenotypic and functional plasticity. Like αβ T cells, γδ T cells also display a Th1, Th2, Th17, and Treg-like phenotype and therefore play an important role in disease progression. Certain subsets of γδ T cells have the ability to secrete interferon gamma (IFN-γ) and IL-4 in a manner similar to Th1 and Th2 cells.

IL-17 production by γδ T cells appears to have an advantage over IL-17 production by αβ T cells in early immune responses because it is potent and rapid and does not require an antigen-specific primer or clonal expansion.

γδ T cells interact with other innate and adaptive immune cells and modulate their function. γδ T cells, through the production of proinflammatory cytokines, help B lymphocytes produce autoantibodies and participate in the pathogenesis of autoimmune diseases [10].

Additional evidence has shown that γδ T cells facilitate differentiation of the monocyte/macrophage lineage. Different γδ T cell subsets play opposing roles in macrophage homeostasis, demonstrating the complexity and plasticity of γδ T cells [5].

The predominant distribution of γδ T cells in mucosal and epithelial tissues makes them the first line of defense against the external environment, for example, allergens; they contribute to immune surveillance and participate in the initiation of mucosal inflammation. Therefore, γδ T cells are believed to act as a bridge between innate and adaptive immunity. By producing Th2-type cytokines (IL-4, -5, and -13), they intensify allergic inflammation of the airways [19].

γδ T cells can participate in fighting infection in many ways. One is to act as antigen-presenting cells (APCs) and promote the recruitment of effector cells to the site of infection. γδ T cells have been shown to facilitate bacterial clearance by recruiting neutrophils, macrophages, and natural killer cells and also contribute to the production of IFN-γ at the site of infection [3].

Lockhart et al. demonstrated that γδ T cells in the lung produce IL-17 after *Mycobacterium tuberculosis* infection, confirming the relationship between γδ T cell activation, neutrophil recruitment, and resolution of the infection. Indeed, this study showed that despite the relatively low percentage of γδ T cells in the lymphocyte compartment, these cells are a more potent source of IL-17 compared with activated CD4+ T cells. The number of γδ T cells producing IL-17 is also increased in patients with active pulmonary tuberculosis [20].

Interleukin-17 plays a protective role against bacterial and fungal infections by recruiting neutrophils, activating T cells, and inducing antimicrobial peptides and inflammatory cytokines. γδ 17 cells produce significantly more IL-17 than Th17 cells following infection with *Mycobacterium tuberculosis* or *Mycobacterium bovis* BCG. γδ 17 cells produce IL-17 rapidly when infected with fungi such as *Candida albicans*. γδ 17 cells are observed in the lung after systemic *Candida albicans* infection. Due to the production of IL-17 by γδ T cells in the early stage of infection, it is suggested that γδ 17 cells play an important role in the early host defense against the development of adaptive immunity [21].

Observations in mice and humans indicate that γδ T cells may be important for the cellular immune response in early ontogeny when αβ T cells responsible for acquired immunity are not yet fully developed [22].

γδ T cells monitor skin integrity by recognizing damaged cells and producing fibroblast growth factor-7 (FGF-7) and insulin-like growth factor-1 (IGF-1). Activated T cells residing in the skin accelerate wound healing. Immune cells, including epidermal DETCs and dermal γδ T cells, regulate wound healing by secreting cytokines and chemokines that promote re-epithelialization after injury [8].

In addition, γδ T cells play a role in the supervision of the tumorigenesis process. They secrete cytotoxic molecules including perforins and granzymes. Human-skin-derived γδ T cell clones exhibit a cytotoxic response against melanoma cell lines [1]. Human skin γδ T cells express the NKG2D receptor, which stimulates cell lysis. Upon activation, skin-derived γδ T cells produce perforin and induce Fas-mediated cytotoxicity. In cancer patients, the percentage of γδ T cells in peripheral blood is lower than in healthy people [23]. There is a hypothesis proposed by Zocchi that these cells are attracted by tumor antigens and expand to the tumor site, and thanks to their cytolytic abilities, they act as a first-line anticancer defense [24].

Moreover, γδ T cells expressing Vγ2Vδ2 TCR have the ability to recognize phosphoantigens that are important products of microbes such as *Mycobacterium tuberculosis*. This suggests that antigen recognition γδ T cells among varies depending on population and is probably unique to where they reside [9].

## 4. The Role of γδ T Lymphocytes in Autoimmune Diseases

T lymphocytes play an essential role in providing an effective immune response, but if not regulated, they can be harmful [9].

IL-17A plays a key role in the development and progression of autoimmune diseases. Although the main source of IL-17A is the Th17 CD4+ cell population, when autoimmune disorders occur, γδ T cells also contribute to the production of IL-17A. Interestingly, in patients with autoimmune liver disease, such as autoimmune hepatitis, primary sclerosing cholangitis, or primary biliary cirrhosis, a significant increase in the number of γδ T cells in the peripheral blood and liver is observed, confirming their role in autoimmune processes [6].

Growing evidence indicates that inappropriately activated γδ T cells may drive the pathogenesis of autoimmune diseases such as psoriasis, vitiligo, autoimmune hepatitis, autoimmune thyroid disease, rheumatoid arthritis, inflammatory bowel disease, multiple sclerosis, and lymphomas [10,25].

An increased amount of T cells residing in the skin has also been observed in people with alopecia areata. In contrast, a reduction in γδ T cell infiltration and function has been demonstrated in patients with type 2 diabetes and melanoma [9].

In the remainder of this review, we summarize published data describing the role of γδ T cells in psoriasis as an example of an autoimmune disease in which the role of γδ T cells has been extensively studied.

## 5. The Role of γδ T Cells in Psoriasis

Psoriasis is a chronic, relapsing, inflammatory skin disease that affects 1–2% of the world’s population, with an equal gender distribution [26,27]. It is characterized by excessive proliferation of epidermal cells, cutaneous vascular hyperplasia, and a chronic inflammatory process of the skin that is predominantly T lymphocytes [28]. T lymphocytes found in psoriatic lesions include helper CD4+ (Th), cytotoxic CD8+ (Tc), natural killer cells (NK), NK-T, and regulatory (Treg) cells. CD4+ lymphocytes predominate in the dermis and CD8+ predominate in the epidermis. The pathogenesis of psoriasis involves the interaction between T cells (CD8 + Tc1 and CD8+ Tc17, CD4 +: Th1, Th17, and Th22) and skin dendritic cells. IL-6, IL-12, and IL-23 cytokines released by dendritic cells promote Th1, Th17, and Th22 along with Tc1 and Tc17 responses. Psoriasis is currently considered a Th1/Th17/Th22-mediated disease.

Activated dendritic cells (DC) transform into mature antigen-presenting cells and are capable of producing transforming growth factor-beta-1 (TGF- β1), IL-23, and IL-12, which interact with naive T cells.

Proinflammatory cytokines such as IL-23, IL-17, and γδ T cells play an important role in the pathogenesis of psoriasis. IL-23 is produced by DC and macrophages in the skin. This cytokine binds to its receptor (IL-23R) located on cutaneous γδ T cells, which synthesize significant amounts of IL-17, which is subsequently responsible for the progression of psoriasis.

**Figure 1:**
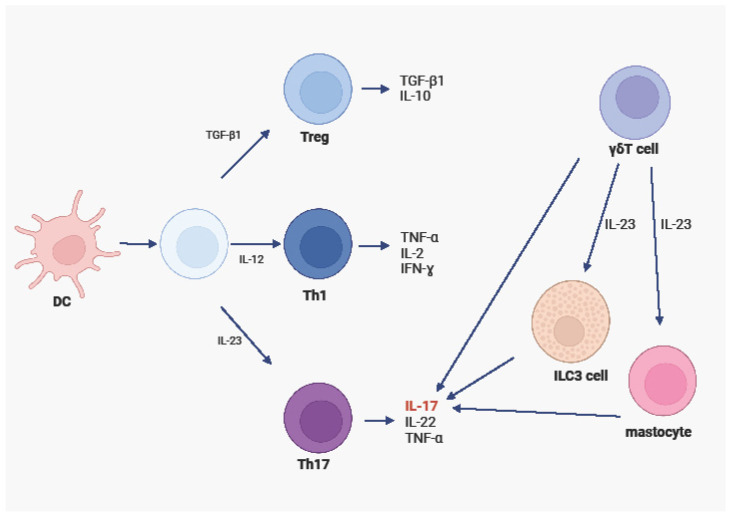
Th1, Th17, and Treg differentiation and their role in the pathogenesis of psoriasis. Own design based on [29,30]; figure created with BioRender.com.

ILC3 cells are a subset of ILCs that have been found in large numbers in psoriatic lesions and in the serum of psoriasis patients. These cells, under the influence of IL-1β and IL-23 stimulation, produce -IL-17 and IL-22, which in turn constitute an important element in the pathogenesis and development of psoriasis [29,30] [Figure 1].

The key role in the induction of psoriatic lesions is played by the disturbance of innate and acquired immunity mechanisms [5]. Up to 90% of lesions appear in areas originally affected by psoriasis, which suggests the presence of an immune memory in the skin [31].

**Figure 2:**
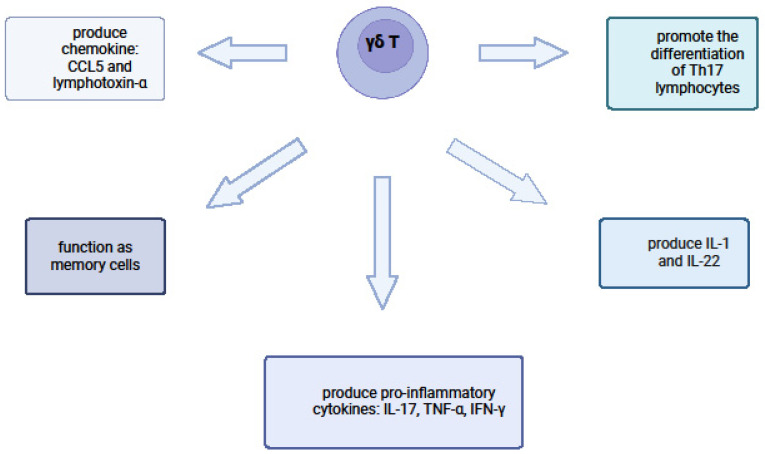
The role of γδ T cells in psoriasis. Own design based on [29,32]; figure created with BioRender.com.

Recent research on psoriasis has focused on the identification of new subpopulations of T cells involved in the pathogenesis of psoriasis, including γδ T cells [32] [Figure 2].

### 5.1. Proinflammatory Cytokine Production

During disease development, γδ T cells produce IL-1 and IL-22, which promotes the differentiation of Th17 lymphocytes. They also produce proinflammatory cytokines: IL-17, Tumour Necrosis Factor alpha (TNF-α), IFN-γ, and a chemokine for T lymphocytes: CCL5 and lymphotoxin-α [29,30].

It was shown that the skin of psoriasis patients had an increased level of Vγ9Vδ2 T lymphocytes compared with the healthy control group. However, a reduction in the number of circulating Vγ9Vδ2 lymphocytes was observed in the blood of psoriasis patients. The number of Vγ9Vδ2 T cells in the blood returned to normal levels after successful treatment of psoriasis.

Vγ9Vδ2 T cells have been shown to produce cytokines (IFN-γ, TNF-α, and IL-17A) and chemokines (IL-8, CCL3, CCL4, CCL5, and CCR6) responsible for recruiting immune effector cells to the skin for activation of keratinocytes [5].

These cytokines lead to the recruitment of more lymphocytes, neutrophils, and bone marrow cells, creating a positive feedback loop that keeps the skin inflamed [9].

The main factor stimulating the activity of γδ T cells in psoriatic skin is Il-23, produced by macrophages, dendritic cells, and Langerhans cells. This process is also regulated by IL-1β, which enhances IL-23-induced activation and proliferation of γδ T cells [33].

The cytokines IL-1, IL-6, IL-18, and TGF-β are also involved in promoting IL-17 production by γδ T cells.

As already mentioned, IL-17 production by cutaneous γδ T cells requires stimulation by endogenous IL-1β.

IL-1α or IL-1β in synergy with IL-23 has been shown to play a key role in the induction of IL-17 from γδ T cells in both mice and humans [3].

IL-1β activates the mTOR signaling pathway through IL-1R-MyD88, while IL-23 activates the STAT3 pathway.

The importance of γδ T cells was also demonstrated in a mouse study using imiquimod (IMQ). It was observed that epidermal hyperplasia and inflammatory response induced by IL-23/IMQ were significantly reduced in γδ T cell-receptor-deficient mice; however, no significant changes were observed in β T cell-receptor-deficient mice [5].

### 5.2. γδ T Lymphocytes as Memory Cells

Notably, γδ T cells have the characteristics of memory cells that respond rapidly to secondary stimulation. This contributes to psoriasis flare-ups.

Recent studies of the etiopathogenesis of psoriasis have shown that after the disappearance of psoriatic plaques in healthy skin, traces of inflammation can still be found in the form of tissue-resident memory (TRM) cells [25].

Insight into TRM cells in various tissues has mainly been gained from studies in mice [34]. TRM cells are able to initiate an inflammatory cascade and cause the recurrence of lesions at the same site. In psoriasis, even in remission, they become a source of important inflammatory cytokines such as IL-17A and IL-22 [31].

TRMs are a subpopulation of memory T cells that persist in nonlymphoid peripheral tissues for long periods of time without recirculating into the blood, thus providing the first line of adaptive cellular defense by acting as alarm or cytotoxic cells. They can quickly cause inflammation and thus relapse [35].

CD69 and CD103 (integrin αE) are key surface markers of TRM cells. CD103 is an E-cadherin ligand that is expressed in epithelial cells, and CD69, a C-type lectin, is expressed in most TRM cells. CD69 is supposed to act as a signal that prevents TRM from leaving tissues by blocking the sphingosine-1-phosphate receptor 1 (S1PR1) [36].

CD8(+) CD69(+) CD103(+) TRM cells are a well-characterized subtype that develops in the epidermis. Local mediators such as interleukin (IL)-15 and TGF-β are required to form a population of long-lived TRM cells in the skin [27]. TRM cells in the skin play a key defense role against skin infections. In addition to this essential physiological role, they are also involved in pathological conditions. The functions of these cells appear to vary depending on each pathology. Psoriatic plaques are visible in a recurrent way, especially in the originally affected places. Upon stimulation of the skin of patients with psoriasis, CD8(+) CD103(+) CD49a(−) TRM cells in the epidermis appear to be reactivated and initiate IL-17A production [36].

## 6. Treatment of Psoriasis Targeting γδ T Cells

γδ T cells and their associated molecules have become targets for new drug development.

One of them is adiponectin, a mediator of insulin sensitivity, playing a key role in metabolic regulation and inflammatory processes. Adipose tissue secretes many bioactive molecules called adipokines, such as adiponectin, leptin, omentin, IL-1β, and TNF-α, which play proinflammatory and anti-inflammatory roles in the metabolic syndrome [17].

Studies have shown that the infiltration of IL-17-producing γδ T cells in psoriatic lesions was significantly increased in the absence of adiponectin. The negative regulation of adiponectin on IL-17 production by dermal γδ T cells is mainly mediated by the AdipoR1 receptor. Increasing adiponectin levels may be effective in treating psoriasis.

BTLA (B and T lymphocyte attenuator) belongs to the immunoglobulin superfamily and has been found to play a role in γδ T cell homeostasis in lymphoid tissues and controls IL-17 production in lymph nodes. The anti-BTLA agonist antibodies have been shown to inhibit γδ T cell expansion and IL-17 production in lymph nodes and skin induced by imiquimod. Thus, BTLA may be a potential target for the treatment of psoriasis.

Indirubin is a bisindole compound extracted from the leaves of the Chinese herb *Indigo naturalis*. It has been shown to ameliorate imiquimod-induced dermatitis by reducing inflammatory responses mediated by IL-17A-producing γδ T cells by activating Jak3/Stat3.

Cutaneous γδ T cells constitutively express the CCR6 receptor. CCR6KO or anti-CCL20 monoclonal antibodies administered to mice reduced IL-23-induced dermatitis. This shows that CCL20 along with its receptor CCR6 are potential targets for the treatment of psoriasis [5].

Nguyen et al. showed that nonresident γδ T cells regulate the expansion of a CD11b (+) Ly6G (+) neutrophil population and their recruitment to joint and skin tissues, where they develop hallmark pathologic features of psoriatic arthritis. The role of γδ T cells in neutrophil regulation can be exploited therapeutically in psoriatic arthritis patients [37].

Monocytes from psoriasis vulgaris patients express higher levels of the BTN3A1 than monocytes from healthy controls. Blockade of BTN3A1 suppresses Vγ9Vδ2 T cell activation and abolishes the difference in Vγ9Vδ2 T cell activation between psoriasis vulgaris patients and healthy controls. The results indicate that BTN3A1 may be a potential target for the treatment of psoriasis [38,39].

IL-23 and IL-17 are involved in the pathogenic function of IL-17-producing cells including Th17 cells and dermal γδ T cells. There are a couple of commercial monoclonal antibodies targeting IL-23p40, IL-17, or IL-17R in clinical studies, which all have shown remarkable therapeutic efficacies towards the treatment of psoriasis.

Because IL-17-producing γδ T cells may be the primary producers of IL-17 in murine models of psoriasis, therapies targeting IL-17 have been approved or are in clinical trials and have proven effective in the treatment of psoriasis [8].

Il-17 inhibitors available for the treatment of psoriasis include secukinumab, ixekizumab, and brodalumab. Il-23 inhibitors include ustekinumab, guselkumab, Risankizumab, and tildrakizumab.

Inhibition of IL-17 signaling effectively treats symptoms in both psoriasis patients and imiquimod-induced psoriasis models, supporting the important role of γδ 17 cells in both humans and mice.

In recent years, several antibodies targeting IL-17 and its receptor have been approved. Secukinumab, an anti-IL-17 antibody, was approved in Japan in 2014 and by the U.S. Food and Drug Administration in 2015 for the treatment of psoriasis and psoriatic arthritis. Treatment of psoriasis patients with secukinumab in a phase III trial showed that more than half of patients achieved near-complete remission after 12 weeks of treatment, as measured by the Psoriasis Area and Severity Index (PASI 90). Additionally, secukinumab has also been approved for the treatment of ankylosing spondylitis. Ixekizumab, a monoclonal antibody against IL-17, and brodalumab, a monoclonal antibody against IL-17RA, are also effective in the treatment of psoriasis in phase III trials and are approved for the treatment of psoriasis [21].

Psoriatic lesions recur in the same area after discontinuation of treatment.

TNF- α, IL-12/23 and IL-17 inhibitors have been shown to have strong and rapid therapeutic efficacy. However, these biological agents have been associated with several side effects, such as increased susceptibility to infections. Long-term safety concerns and high costs hinder the widespread use of these agents [40].

## 7. Comorbidities in Psoriasis

Psoriasis is accompanied by many well-known comorbidities, in particular psoriatic arthritis, depression, cardiovascular diseases, type II diabetes, metabolic syndrome, including abdominal obesity, atherosclerosis, hypertension, nonalcoholic fatty liver disease, gastrointestinal diseases, Crohn’s disease, kidney diseases, and osteoporosis [41].

The pathogenesis of comorbidities remains unknown. Common inflammatory pathways and cellular mediators may likely be among the factors [42]

### 7.1. Depression and Psoriasis

Psoriasis is a disease that also affects the patient’s mental state. The co-occurrence of depression in psoriasis may be partially attributable to elevated levels of proinflammatory cytokines, rather than simply the psychosocial impact of the disease itself [42]. It is a disorder accompanied by cellular immune activation and inflammation. The key regulators of these pathophysiological processes are T cells and numerous proinflammatory cytokines, including TNF-α, IL-1β, IL-2, IL-6, IL-12, IL-17, IL-18, and IFN-γ. Several studies have shown a strong association between depression and psoriasis exacerbation. This was manifested by increased concentrations of proinflammatory cytokines in patients with depression and psoriasis and reduced symptoms of depression in patients with psoriasis treated with anticytokines [43].

Marek-Józefowicz et al. demonstrated a significant incidence of depressive symptoms in patients with psoriasis. More than 50% of respondents had moderate to severe depression. This clearly indicates that psoriasis and depression can co-occur. The severity of depression is associated with a poorer quality of life in patients. There are more and more reports highlighting the immunological hypothesis of depression. Studies of common markers of both disorders emphasize the abnormal function of the stress axis, with particular importance attached to interleukin-1 (IL-1), interleukin-6 (IL-6), and cortisol. Several studies have shown that inflammatory–immune processes play an important role in the course of depression and probably play a role in the regulation of the hypothalamic–pituitary–adrenal axis [44,45,46].

The intestinal immune system interacts with the commensal microflora. Moreover, stress changes the composition of the microbiome, leading to impaired brain function. A reduction in specific *Lactobacillus* species contributes to stress-induced social avoidance behavior observed in depressed patients.

Zhu et al. demonstrated that colonic γδ T cells modulate behavioral susceptibility to chronic social stress through dectin-1 signaling. Stress behaviors result from increased differentiation of Il-17-producing γδ T cells in the colon and their accumulation in the meninges. These stress-prone cellular and behavioral phenotypes are causally mediated by dectin-1, an innate immune receptor expressed on γδ T cells. The results highlight the importance of dectin-1 as a potential therapeutic target for the treatment of stress-induced behaviors [47].

### 7.2. The Role of γδ T Cells in Obesity

Many epidemiological studies have confirmed the relationship between obesity and psoriasis, and it is believed that obesity is an independent risk factor for its development and is associated with a worse prognosis [48].

Herron et al. showed that the prevalence of obesity in the study population of psoriasis patients was higher than in the general Utah population (34% vs. 18%; *p* < 0.001) [49].

Obesity is a condition in which the patient reaches a BMI above 30 kg/m^2^ [42]. Adipose tissue is an essential endocrine organ secreting a wide range of mediators involved in immune mechanisms, inflammation, and the regulation of metabolism and appetite. Proinflammatory cytokines produced by visceral adipose tissue, such as TNF-α, IL-6, IL-17, and adipokines (adiponectin, omentin, chemerin), participate in the development of dyslipidemia, insulin resistance, diabetes and, consequently, cardiovascular diseases [48].

Leptin, an adipocyte-derived hormone, as an inflammatory mediator, stimulates the production of chemokines IL-1β and TNF-α from blood monocytes in obese patients with psoriasis. Increased serum leptin concentration in patients indicates its significant role in the pathogenesis of psoriasis. Multilevel treatments are recommended for obese patients with psoriasis to treat obesity-induced inflammation. Controlled weight loss through exercise and diet has shown improvement in the clinical condition of obese patients with psoriasis [43]. Therefore, treatment of patients with psoriasis should also include education about healthy eating habits and body weight monitoring.

The proinflammatory state generated by obesity may be a key factor in the link between obesity and inflammatory/autoimmune diseases [50]. Obesity leads to chronic systemic inflammation. It is associated with various metabolic disorders such as diabetes, insulin resistance, and cardiovascular diseases [51].

In pathological conditions, including obesity, dysfunctional adipose tissue functions as antigen-presenting cells, which makes it susceptible to infiltration by specific immune cells. Immune cells, including ILCs, natural killer cells, leukocytes, and lymphocytes, accumulate in adipose tissues to produce an immune response. In turn, macrophages and T cells residing in adipose tissue promote ROS production along with the expression of cytokines and chemokines (TNF-α, IL-6, IL-10, and IFN-γ) to enhance the inflammatory response [43].

γδ T cells have been suggested to play a key role in chronic inflammatory diseases. Recently, it has been observed that γδ T cells are also present in adipose tissue [52]. It was found that obese patients had a reduced level of V γ 9V δ 2 T lymphocytes and the level of IFN-γ secreting [51]. The reduction in V γ 9V δ 2 T cells was inversely related to body mass indexes [53]. Studies in mice have shown similarly reduced numbers of γδ T cells in the skin of obese mice [53].

In obesity and metabolic diseases, there is a reduced or altered level of growth factors at the wound site, which impedes leukocyte infiltration, cell growth, and migration through the wound. Studies show that in obese mice, the number of keratinocytes is reduced, and the epidermis has a flattened and thinner appearance, supporting the hypothesis that dysfunctional γδ T cells contribute to impaired keratinocyte homeostasis in obesity. In addition to dysfunctional γδ T cells, keratinocyte homeostasis is further exacerbated by factors associated with obesity, as evidenced by altered E-cadherin localization and impaired cell adhesion.

This affects the integrity of the epithelium in the case of obesity and increases the susceptibility of the epidermis to damage, environmental factors, and pathogens [54].

Obesity is associated with increased susceptibility to both viral and bacterial pathogens. Costanzo et al. found that the number of Vγ9Vδ2 T cells is reduced in the peripheral blood of obese donors. However, the remaining Vγ9Vδ2 T cells in obese donors show increased differentiation towards the effector T memory population and an abnormal effector response to influenza infection. Obesity has a negative impact on both the number of cells producing IFN-γ and the amount of IFN-γ produced per cell, which reduces the antiviral response.

Vγ9Vδ2 T cell dysfunction in obesity can be reversed by the addition of IL-2 signaling during influenza infection. Obesity is associated with reduced numbers of Vγ9Vδ2 T cells, while αβ T cells persist or even increase. Vγ9Vδ2 T cells from obese donors express similar levels of NKG2D and intracellular granzyme B to lean donors, suggesting that these key cytotoxicity mechanisms are conserved [55,56,57,58].

**Figure 3:**
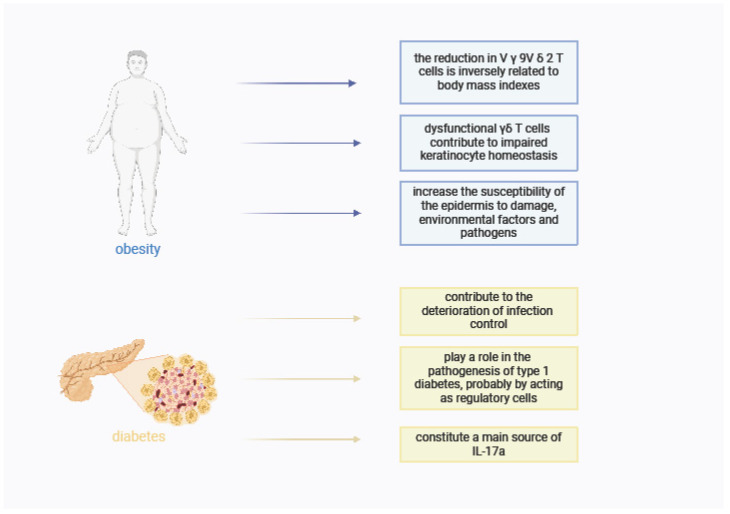
The role of γδ T cells in comorbidities in psoriasis. Own design based on [55,56,57,58]; figure created with BioRender.com.

Figure 3 shows the role of gamma delta T cells in diseases comorbid with psoriasis—diabetes and obesity [Figure 3].

### 7.3. The Role of γδ T Cells in Diabetes

In people with type 2 diabetes, hyperglycemia causes disturbances in the proliferation of skin T lymphocytes, which results in a reduction in the number of T lymphocytes in the epidermis. These changes likely contribute to the development of chronic, nonhealing wounds and persistent infections observed in obese patients.

T *γδ* cells have altered metabolic pathways, which makes them unresponsive to damage to epithelial cells. γδ T cells contribute to the deterioration of infection control observed in the obese diabetic population [52]. γδ T cells are the main source of IL-17a, a cytokine that regulates adipogenesis and glucose metabolism in adipose tissue. Studies have shown that IL-2 is able to restore the production of antiviral cytokines by γδ T cells [55].

Type 1 diabetes is an organ-specific autoimmune disease in which activated, autoreactive T cells damage insulin-secreting β-cells in the pancreas. Type 1 diabetes is characterized by the infiltration of innate and adaptive immune cells in the pancreatic islets called insulitis [10].

Furthermore, in islet biopsies from patients with diabetes, γδ TCR sequences predominated among the identified T cell clonotypes, suggesting that γδ T cells infiltrate pancreatic islets in patients with type 1 diabetes. These data suggest that γδ T cells may participate in the pathogenesis of type 1 diabetes [11].

O’Brien et al. showed that in mice with type 1 diabetes, some γδ T cells actively promote the development of the disease, while others protect against it. The results further suggest that γδ T cells influence diabetes in mice, at least in part, by inducing changes in the levels of other T cells, including CD4+ Tregs, as well as other γδ T cell subsets. In human type 1 diabetes, γδ T cells may similarly influence the development of diabetes [59].

Zubkiewicz-Kucharska et al. demonstrated that the γδ CD8+ T subset plays a role in the pathogenesis of type 1 diabetes, probably by acting as regulatory cells. Therefore, the decrease in these lymphocytes makes the studied patients more susceptible to autoimmunity, including type 1 diabetes. No relationship was found between the γδ T cell population and age. However, in patients with type 1 diabetes, a negative correlation was found between age and T γδ CD4+CD8– and T γδ CD69+, and a positive correlation was found between age and T γδ CD56+ [23].

Thus, these studies suggest that γδ T cells play an important role in the pathogenesis of autoimmune diabetes, and a better understanding of their molecular mechanism will enable the development of effective methods to control autoimmunity.

Lang et al. showed that the development of an abnormal response to the intravenous glucose tolerance test and progression to diabetes were associated with a reduction in the percentage of γδ T cells. However, patients whose percentage of γδ T cells remained high retained normal β-cell function. Data suggest that γδ T cells are involved in the autoimmune process leading to diabetes and may play a regulatory role. Monitoring their levels in the blood of patients at risk of diabetes may be useful as an additional prognostic factor for the development of diabetes [60].

## 8. The Role of γδ T in Atopic Dermatitis

Increased expansion and activation of γδ T cells in the skin is a common feature of acute and chronic inflammatory skin conditions such as psoriasis, contact dermatitis, and atopic dermatitis [17].

Atopic dermatitis (AD) is a chronic, relapsing inflammatory skin disease mediated by type 2 T cells (Th2). Human epidermal γδ T cells can produce IL-4 and IL-13, the main Th2 cytokines, suggesting their role in the pathogenesis of atopic dermatitis [61].

Recent research suggests that not only αβ T cells but also the less common γδ T cells may play a role in the development and maintenance of allergic inflammation.

Cairo et al. investigated the role of γδ T cells in atopic dermatitis. The percentage of circulating Vγ 9Vδ2 + T cells was significantly increased in patients with atopic dermatitis compared with age-matched controls. They showed that there was an association between circulating γδ T cell levels and atopic dermatitis, with a positive correlation with clinical outcome but no association with serum IgE levels [62].

Svensson et al. demonstrated that Τ γδ cells promote allergic airway inflammation by enhancing the systemic IgE response and local antibody reactivity without a specific role in shifting the immune response towards Th2 [63].

In children with AD, a significant increase in the frequency of Vγ9Vδ2 lymphocytes is observed, which highlights the positive correlation between their expansion and the severity of the disease. This increase may be due to the typical presence of Staphylococcus aureus in the skin of AD patients [17].

The number of IL-17A-producing cells is increased in peripheral blood mononuclear cells in patients with severe AD, and cutaneous γδ T cells in the skin were the main sources of IL-17A.

Nakajima et al. investigated whether the spontaneous development of dermatitis, Il-4 production, and IgE induction was attenuated by IL-17A deficiency. As a result, it appears that IL-17A promotes Th2, and IL-4, as a Th2 cytokine, may inhibit IL-17A production through a feedback mechanism. The above data suggest that IL-17A mediates Th2-type immune responses from the perspective of AD development and that IL-17A signaling may be a therapeutic target for AD [64].

The pathophysiological role of γδ T cells in AD requires further research.

## 9. The Role of γδ T Cells in Cancer Processes

T cells play a key role in anticancer immunity. Currently, in addition to αβ T cells, the role of γδ T cells is also noticed in inhibiting cancer processes. It turns out that γδ T cells, after migrating to the tumor site, may have anticancer effects [65].

γδ T cell recognition and response to cancer cells is very different from αβ T cells [14]. The antitumor effect of γδ T cells, unlike αβ T cells, does not depend on the mutational load, which makes them effective in cancers with few somatic mutations. Another important issue is the fact that they do not act in a manner dependent on antigen presentation via MHC I. Moreover, they show increased anticancer activity due to their specific activation mechanisms present in both adaptive cells via TCR signaling and innate cells via NK signaling [65]. Although relatively few antigens have been associated with the recognition of cancer cells by γδ T cells, it is now known that the phosphoantigen sensors BTN3A1 or CD277 play a key role in the TCR-dependent activity of these cells. In addition to the TCR, γδ T cells can utilize various NK cell receptors for their potent cytotoxic functions. Some of the NK cell receptors that may participate in tumor cell targeting by human γδ T cells are NKG2D, DNAM-1, NKp30, and NKp44. Additionally, the activation of DNAX-activating molecule 1 (DNAM-1), leukocyte-function-associated antigen (LFA-1), and costimulatory receptor CD27 receptors leads to T cell activation and increased cytotoxicity [66].

The presence of transformed, infected, or stressed cells may lead to the induction of cell death by γδ T cells. Mechanisms of antitumor action include direct cytotoxicity via perforins, granzymes, and tumor necrosis factor-related apoptosis-inducing ligand receptors. γδ T cells also interact with neutrophils, αβ T cells, dendritic cells, and B cells to trigger an immune response. Moreover, these cells, by producing IFN-γ and IL-17, attract other immune mediators to the site of infection [7].

IFN-γ-producing γδ T cells include fetal DETCs in the epidermis and postnatally generated cells in the dermis and lymphoid tissues. IL-7 promotes the development of γδ T cells, which in turn produce Il-17, while Il-15 and Il-2 induce the secretion of IFN-γ by γδ T cells. Moreover, cytokines such as IL-12 and IL-18 promote the production of IFN-γ, while IL-1β and IL-23 affect IL-17-producing cells. γδ T cells, through the production of proinflammatory cytokines, including IL-17A, IFN-γ, TNF, chemokines (CCL5, CXCL10), and lymphotactin (XCL1), influence the recruitment of neutrophils and macrophages. In this way, they regulate many immune responses. γδT1 cells, which produce IFN-γ, mainly play an anticancer function. The population of γδ1 + T cells producing IFN-γ is considered a positive prognostic marker in cancer. IFN-γ-producing γδ cells are potent type 1 antitumor cytotoxic effectors, whereas Il-17-secreting γδ T cells are mainly protumor due to the induction of angiogenesis and cancer cell proliferation [8].

Gao et al. showed that mice with IFN-γ-deficient γδ T cells have a higher incidence of tumors than those that have IFN-γ-secreting γδ T cells. γδ T cells, through the production of IFN-γ, have been shown to regulate MHC class I expression on murine B16 melanoma cells, thereby promoting their recognition by CD8+ T cells.

NK, NK T, and γδ T cells, which have characteristics of both innate and adaptive immune cells, may be the initial source of IFN-γ in protection against cancer. This, in turn, may facilitate the adaptive immune response cascade and increase subsequent IFN-γ production [13].

The above characteristics of γδ T cells make them very attractive for use in anticancer therapy.

### 9.1. Therapeutic Strategies

Taking into account the strong anticancer effect of γδ T cells, various methods are being sought to increase their cytotoxic properties. A subset of Vγ9Vδ2 cells in humans recognizes cancer cells primarily by increasing the expression of phosphoantigens. There are several methods to increase cancer cell recognition by Vγ9Vδ2 cells. One of them is the strengthening of the BTN3A1/BTN2A1 complex activated by IPP. Bisphosphonate drugs increase the accumulation of PPIs, making tumor cells more susceptible to killing Vγ9Vδ2 cells, but these drugs also induce the proliferation of Vγ9Vδ2 cells in culture. γδ T cells are also equipped with chimeric antigen receptors in hematological and epithelial malignancies [14]. In the context of cancer treatment, it is important how commonly used drugs can affect the activity of γδ T cells. Low doses of commonly used chemotherapy drugs such as 5-fluorouracil, doxorubicin, and cisplatin sensitize colon cancer cell lines to Vγ9Vδ2 T cell cytotoxicity. Decitabine, a drug that inhibits DNA methylation, increases the levels of NKG2D ligands in osteosarcoma cell lines and enhances their targeting by Vγ9Vδ2 T cells. However, when γδ T cells alone are exposed to decitabine, their proliferation and cytotoxic properties are attenuated [67]. There are several hurdles that must be overcome before γδ T cells can be effectively used in cancer therapy. An appropriate way to deliver γδ T cells to solid tumors must be developed by using mechanisms that direct γδ T cells to assume an antitumor rather than a protumor function. It would also be important to discover ligands that activate and expand specific γδ T cell populations [68].

Most γδ T cells recognize target cells regardless of HLA antigen presentation. Therefore, allogeneic-donor-derived γδ T cells may be relatively safe due to the low risk of graft-versus-host disease (GvHD). Leukemia patients who underwent allogeneic αβ-deficient stem cell transplantation from partially HLA-incompatible donors showed longer 5-year and overall survival, reduced risk of relapse, and reduced risk of GvHD. Some of these studies have already been completed, and no serious side effects have been reported, highlighting the safety of Vγ9Vδ2 T cell transfer [69]. Most strategies currently being evaluated take advantage of the safety of γδ T cell activation and include tumor-targeting mechanisms, for example, chimeric antigen receptor (CAR) or bispecific γδ T-cell engagers (bsTCE) [70]. In recent years, CAR-T cell therapy has been extensively explored in preclinical and clinical trials, mainly focused on conventional αβ T cells. These autologous CAR-T cells have induced encouraging remission rates in patients’ refractory to standard therapies, particularly against B-cell malignancies [71]. Most research on human γδ T cells has focused on the peripheral blood Vγ9Vδ2 T cell subset. This is due to the ease of obtaining cells for laboratory tests and the possibility of cultivating large numbers of cells. The properties of circulating Vδ1 cells are less defined, although they have been studied for the treatment of certain cancers. Preliminary results of therapeutic strategies based on Vγ9Vδ2 and Vδ1 T cells, as well as antibodies, are very promising and confirm their safety. Vγ9Vδ2 T cells are particularly attractive for cancer therapy because they constitute the largest group of memory T cells that respond to a single antigen. Local treatment delivered at the tumor site may be one way to increase Vγ9Vδ2 T cell potential. Many researchers are developing immunotherapies based on the selective activation of the Vγ9Vδ2 T cell subset [72]. However, strategies based on these cells pose challenges such as high cost and difficulty in production. Also, it is worth noting that γδ T cells lack adaptive specificity and proliferative potential against a specific cognate antigen, which limits their use in cellular immunotherapy. Some virus-dependent cancers, such as Burkitt’s lymphoma caused by Epstein–Barr virus infection, can render tumor cells insensitive to γδ T-cell-dependent lysis [73].

The balance between γδ T cells producing IFN-γ and those producing IL-17 in the tumor microenvironment may have a strong influence on their therapeutic effect. Therefore, upcoming clinical trials should focus on activities that promote γδ T cells, which produce IFNγ [67].

### 9.2. Protumor Effect of γδ T Cells

Despite some studies showing that the presence of γδ T cells among tumor-infiltrating lymphocytes is a positive prognostic factor, it is still not fully known whether they promote or inhibit tumor growth.

This dual role may be linked to γδ T cell plasticity, which includes the ability to differentiate into different functional cell subsets depending on the microenvironment. Indeed, after recruitment to the tumor site, under the influence of cytokines, they can acquire a phenotype reflecting Tfh, Treg, Th1, Th2, and Th17 cells. It has been reported that polarized γδ T17 cells and γδ Treg cells, which produce IL-17A or TGF-β, respectively, promote the progression of gallbladder, ovarian, colorectal, and rectal cancers. Therefore, γδ T cells may create an immunosuppressive microenvironment to inhibit antitumor immune responses, evade immune surveillance, and promote cancer progression [74].

## 10. γδ T Cells and Lymphomas

γδ T-cell lymphomas are aggressive and rare hematologic malignancies that develop through the transformation of mature γδ T cells and include hepatosplenic γδ T-cell lymphoma (HSGDTL) and primary cutaneous γδ T-cell lymphoma (PCGDTL). PCGDTL accounts for less than 1% of all primary cutaneous lymphomas but is highly aggressive and associated with high mortality [67]. These tumors originate from γδ T cells, which naturally play a role in the innate, nonspecific immune response. Patients usually present with disseminated skin lesions in the form of ulcerative-necrotic plaques and nodules, mainly located on the limbs. Mucous membranes and other extranodal sites are often involved. Quite common general symptoms include fever, night sweats, and weight loss. Due to its rarity, there are no clinical trials on the treatment of PCGD-TCL. Treatment guidelines are mainly based on case reports. The most commonly used treatment regimen remains the anthracycline-based regimen. The overall prognosis is very poor due to aggressiveness and resistance to chemotherapy [75].

In B-cell malignancies, such as B-cell lymphoma, chronic lymphocytic leukemia (CLL), or multiple myeloma (MM), cancer cells can be found both in the peripheral blood and in lymphatic organs such as the bone marrow and lymph nodes. Malignant cells may interact with other cell types, creating a specific microenvironment in which infiltrating γδ T cells may play an important role. Hodgkin lymphoma patients were shown to have slightly higher levels of circulating γδ T cells compared with healthy donors. γδ T-cell acute lymphoblastic leukemia (γδ T-ALL) arises from the transformation of immature γδ thymocytes and has clinical features that differ from αβ T-ALL [67].

In B-cell non-Hodgkin lymphomas (B-NHL), the main subtypes of circulating γδ T cells have been shown to be Vγ1, Vδ1, and Vδ2. Vδ1 and Vδ2 T cells have been described to contribute to antitumor responses in B-cell malignancies, sometimes in different proportions and with different modes of action. In diffuse large B-cell lymphoma (DLBCL), an aggressive form of B-NHL, γδ T cells constitute a significant population among the infiltrating T cells [65].

γδ T cells facilitate targeted cell death by helping to clear tumors and pathogens while releasing immunomodulatory cytokines such as IFN-γ, IL-17, and TNF-α. The unique characteristics of γδ T cells make them an attractive tool for the development of anticancer therapeutics. Hematologic malignancies, including leukemias, lymphomas, and myeloma, could be targets for cell therapy due to direct access to tumor cells through the vascular and lymphatic systems. Additionally, these tumors typically have a less immunosuppressive tumor microenvironment compared with solid tumors, further increasing the potential for therapeutic effects [66].

## 11. Conclusions

γδ T cells constitute an important subset of T lymphocytes. γδ T cells are heterogeneous populations of cells that play a role in inflammation, host tissue repair, clearance of viral and bacterial pathogens, regulation of immune processes, and tumor surveillance. Inappropriately activated γδ T cells may drive the pathogenesis of autoimmune diseases such as psoriasis, vitiligo, autoimmune hepatitis, autoimmune thyroid disease, rheumatoid arthritis, inflammatory bowel disease, multiple sclerosis, and lymphomas. Understanding the immunological pathways in which these cells participate and the role of the cytokines and chemokines they produce provides a chance for more effective treatment and, consequently, achieving long-term remissions in patients with psoriasis. Furthermore, γδ T cells exhibit memory-cell-like characteristics that mediate repeated episodes of psoriatic inflammation. To reduce immune memory, mitigating skin inflammation at an early stage may be key. Collaboration between doctors of many specialties is also important for the early detection and treatment of diseases comorbid with psoriasis.

γδ T cells, through their antitumor properties, ability to act as antigen-presenting cells, and independence from MHC class I-mediated antigen presentation, allow for greater flexibility in tumor targeting compared with their αβ T-cell counterparts. It turns out that γδ T cells may constitute an excellent therapeutic platform in the treatment of hematological and solid cancers. In recent years, groundbreaking progress has been made in the clinical use of γδ T cells. Research on γδ T cells has revealed their diverse functions and therapeutic implications in autoimmune diseases, infections, and cancer. γδ T cells directly recognize and destroy abnormal cells regardless of HLA antigen presentation; therefore, they seem to be a promising cell line in cancer immunotherapy. Moreover, Vγ9Vδ2 T cells constitute the largest group of memory T cells that respond to a single antigen. A better understanding of the metabolism of cancer and immune cells will enable the development of next-generation immunotherapies. New immunotherapies based on γδ T cells are currently being developed.

## Abbreviations

γδ	gamma delta
αβ	alpha beta
MHC	major histocompatibility complex
APCs	antigen-presenting cells
ROS	reactive oxygen species
Syk	spleen tyrosine kinase
FGF-7	fibroblast growth factor-7
IGF-1	insulin-like growth factor-1
NK	natural killer cells
Treg	T regulatory
TGF-β	transforming growth factor beta 1
IMQ	imiquimod
TRM	tissue-resident memory
S1PR1	sphingosine-1-phosphate receptor 1
Il	interleukin
IFN-γ	interferon gamma
TNF-α	Tumour Necrosis Factor alpha
BTLA	B and T lymphocyte attenuator
BTN3A1	phosphoantigen sensor butyrophilin 3A1
PASI	Psoriasis Area and Severity Index
AD	atopic dermatitis
LFA-1	leukocyte function-associated antigen
IPP	isopentenyl pyrophosphate
HSGDTL	hepatosplenic γδ T-cell lymphoma
PCGDTL	primary cutaneous γδ T-cell lymphoma
CLL	chronic lymphocytic leukemia
MM	multiple myeloma
γδ T-ALL	γδ T-cell acute lymphoblastic leukemia
DLBCL	diffuse large B-cell lymphoma
DC	dendritic cells
B-NHL	B-cell non-Hodgkin lymphomas
DNAM-1	DNAX activating molecule 1
CAR	chimeric antigen receptor
bsTCE	bispecific γδ T-cell engagers
